# Tanzania Health Information Technology (T-HIT) System: Pilot Test of a Tablet-Based System to Improve Prevention of Mother-to-Child Transmission of HIV

**DOI:** 10.2196/mhealth.8513

**Published:** 2018-01-15

**Authors:** Sheana Bull, Deborah SK Thomas, Elias C Nyanza, Sospatro E Ngallaba

**Affiliations:** ^1^ Department of Community and Behavioral Health Colorado School of Public Health University of Colorado Anschutz Medical Campus Aurora, CO United States; ^2^ Department of Geography & Environmental Sciences University of Colorado Denver Denver, CO United States; ^3^ School of Public Health Catholic University of Health and Allied Sciences Mwanza United Republic Of Tanzania

**Keywords:** mHealth, decision aids, HIV, healthcare workers

## Abstract

**Background:**

The prevention of mother-to-child transmission (PMTCT) of HIV requires innovative solutions. Although routine monitoring is effective in some areas, standardized and easy-to-scale solutions to identify and monitor pregnant women, test them for HIV, and treat them and their children is still lacking. Mobile health (mHealth) offers opportunities for surveillance and reporting in rural areas of low- and middle-income countries.

**Objective:**

The aim of this study was to document the preliminary impacts of the Tanzania Health Information Technology (T-HIT) system mHealth intervention aimed at health workers for PMTCT care delivery and capacity building in a rural area of Tanzania.

**Methods:**

We developed T-HIT as a tablet-based system for an electronic data collection system designed to capture and report PMTCT data during antenatal, delivery, and postnatal visits in Misungwi, Tanzania. T-HIT was tested by health workers in a pilot randomized trial comparing seven sites using T-HIT assigned at random to seven control sites; all sites maintained standard paper record-keeping during the pilot intervention period. We compared numbers of antenatal visits, number of HIV tests administered, and women testing positive across all sites.

**Results:**

Health workers recorded data from antenatal visits for 1530 women; of these, 695 (45.42%) were tested for HIV and 3.59% (55/1530) tested positive. Health workers were unable to conduct an HIV test for 103 women (6.73%, 103/1530) because of lack of reagent, which is not captured on paper logs. There was no difference in the activity level for testing when comparing sites T-HIT to non-T-HIT sites. We observed a significant postintervention increase in the numbers of women testing positive for HIV compared with the preintervention period (*P*=.04), but this was likely not attributable to the T-HIT system.

**Conclusions:**

T-HIT had a high degree of acceptability and feasibility and is perceived as useful by health workers, who documented more antenatal visits during the pilot intervention compared with a traditional system of paper logs, suggesting potential for improvements in antenatal care for women at risk for HIV.

## Introduction

### Rationale

According to the World Health Organization (WHO), an estimated 36.7 million people globally live with human HIV, and nearly 70% of them reside in sub-Saharan Africa [1]. Although there has been substantial progress in the fight against HIV, we fall short of global goals to eliminate new infections among children and substantially reduce acquired immune deficiency syndrome–related maternal deaths [2]. Tanzania, with an estimated 5.6% HIV prevalence among pregnant women attending antenatal care (ANC) clinics [3], was among 22 countries targeted by the United Nations Global Plan to eliminate new HIV infections in children [2]. The Global Plan sets a target for reducing the mother-to-child transmission of HIV during pregnancy and breastfeeding to less than 5%, although transmission in many countries remains stubbornly high [2]. Key elements of prevention of mother-to-child transmission (PMTCT) include: testing pregnant women for HIV, initiating lifelong antiretroviral (ARV) therapy for women with HIV (called Option B+, promoted via WHO guidelines in 2010) [4], delivering in a care facility, initiating nevirapine therapy for exposed infants, diagnosing infants, and offering ongoing linkages to care.

In Tanzania, the primary challenges include limited ANC clinic attendance across the four recommended WHO visits and limited integration between PMTCT and broader maternal and child health services [5,6]. In sharp contrast to the WHO goals, only an estimated 76% of HIV positive pregnant women attending ANC clinic also received PMTCT services, and only 43% of HIV exposed infants received ARV drugs [5,6]. Although routine monitoring is effective in some areas, we continue to lack a standardized and easy-to-scale solution to identify and monitor pregnant women, test them for HIV, and treat them and their children.

Mobile platforms such as phones and tablets have tremendous potential to impact health care delivery and health outcomes. The use of cell phones worldwide has expanded rapidly over the past decade in both developed and developing countries. By the end of 2015, there were nearly 7.1 billion mobile cellular subscriptions globally, and close to 100% of the population was covered by a mobile signal, a drastic increase from 20% coverage in 2003 [7], even reaching poor-resource settings [8]. The universality of cell phones provides an opportunity for their use in broad and scale-up of technology-based health interventions, particularly in developing and resource-poor areas. A proliferation of innovations that integrate the use of mobile and wireless devices to improve health outcomes, health care services, and health research into care delivery, often called *mHealth*, has occurred concomitantly with the growth of cell phone usage [9].

Researchers have implemented mobile health (mHealth) apps in a range of settings and multitude of health targets [10] for facilitation of care delivery, medical records charting, patient and health worker education, disease prevention, and patient self-management. These tools can improve surveillance, clinical care, prevention, and self-management. Furthermore, they have the potential to expand population-level public health impacts through wider dissemination and scale-up for wide spread use [11]. Successful mHealth interventions can often intensify their effects when they are guided by behavioral and social science theory to help in the design, implementation, and analysis of effects [12].

Although mHealth has more often focused on prevention and self-management for behavior change at the individual level, attention has broadened toward targeting the health care worker in rural and resource-poor settings as a possible sustainable intervention model. Our own recent report offered a synthesis of findings from 31 peer-reviewed studies related to the use of mHealth solutions for delivery of health care in resource-limited settings [13]. Overall, the findings demonstrate a substantial benefit to health workers, their patients, and care delivery systems when mobile technology tools such as mobile phones and tablets are used. Acceptability of these tools for care delivery is high, and evidence shows that the use of mHealth tools can improve communication between health workers and their patients, health workers and clinic staff, as well as between health workers and their supervisors. Use of mHealth tools by health workers is associated with improved compliance with treatment protocols among patients and improved health outcomes. mHealth tools are used successfully in surveillance efforts to improve quality and efficiency of data collection [13].

### Objective

The Tanzania Health Information Technology (T-HIT) system was developed as an easy-to-use tablet-based patient data collection mHealth solution that could potentially be scaled to health facilities throughout the region with the goal of improving the continuity and quality of care for PMTCT delivery to women in a rural, resource-limited setting. In addition to improved and integrated patient records that can enhance continuity of care through the linking of patient records over time, electronic record-keeping also enables the documentation of trends and patterns in overall care delivery. Thus, T-HIT has the potential to enhance care delivery and function as a decision-support tool for health workers and administrators. This paper aims to (1) document T-HIT system use and describe health worker clinical activities at the health facility level for PMTCT, (2) demonstrate T-HIT feasibility for use by health workers over a 3-month period, and (3) document system capacity for HIV testing and identification of new HIV infections.

## Methods

### Study Design

This was a randomized controlled pilot study. Working in Misungwi District in Northwestern Tanzania, we identified fourteen health facility sites within the district that represented a mix of hospital, health centers, and dispensaries from the total of 42 health facilities. The Tanzanian health system, which is structured similar to many other sub-Saharan countries, is organized in a pyramid referral structure, with dispensaries being the smallest, then health centers, district hospitals, and finally regional hospitals. All health facilities in the district were eligible to participate.

To ensure equivalent resource distribution across intervention and control sites, we matched pairs and planned for the intervention and control groups to each have a mix of hospital (one intervention or one control), health centers (two intervention or two control), and dispensaries (four intervention or four control). This includes all hospitals and all health centers in the district and eight of the remaining 36 dispensaries as an intervention or control site in the study. Stratification included (1) Facilities that were closer (less than 20 kilometers) or further (greater than 20 kilometers) and (2) Dispensaries with more prior experience (having PMTCT during 2010 preliminary work) or less prior experience (having no PMTCT during 2010 preliminary work). Once matched, we randomly assigned a facility within each pair to either intervention (training for, and use of, the T-HIT system) or control (standard of care, which is recording visits in handwritten paper logs).

### Development of the Tanzania Health Information Technology System

The focus of the T-HIT system was on strengthening PMTCT through an mHealth tablet-based solution for health workers that acts as a platform for capturing patient data and providing some immediate feedback to health workers to enhance care. To that end, T-HIT was designed with three primary features to support the electronic capture of data relevant for PMTCT outcomes. First, it facilitates data collection during a clinic visit for PMTCT in a way that mimics the data elements captured in paper records. Second, it also serves to help reinforce health worker clinical decisions with integrated alerts and reminders about specific protocols. Finally, a reporting dashboard relays composite data for each health facility and for the district (in this instance all seven facilities) in near real time. With paper records, reporting is much more limited. Importantly, T-HIT interface was designed with feedback from health workers and the district health management team.

**Figure 1:**
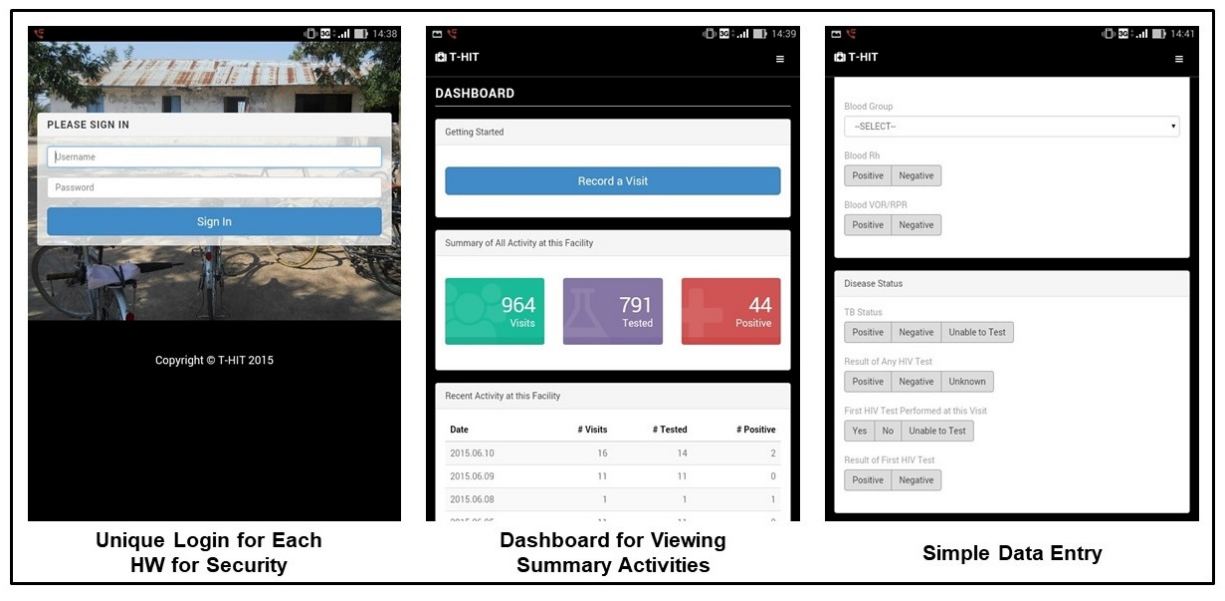
Selected screenshots of the Tanzania Health Information Technology (T-HIT) system.

For this initial pilot, the T-HIT system was designed to document delivery of a subset of elements traditionally documented within an antenatal face-to-face visit for a mother with, or at risk of HIV, and would typically be recorded in paper logs maintained at the health facility and on the maternal and child health (MCH) card that a woman keeps with her. Because the goal was, in part, to ensure compatibility with current work flows, nearly no changes in data elements were made to those collected and charted within the system compared with the paper standard of care. The T-HIT interface was designed to capture these data elements with ease-of-use in mind, using clean and uncluttered interfaces with drop-down menus and check box options to limit errors in data entry (see Figure 1).

### Data Collection

An inventory of all facilities in the district was conducted in summer 2014 and updated in January 2015 to establish cell phone connectivity and availability of electricity, as well as to develop base maps of facilities and roads for use in descriptive mapping of the data across the district. All facilities in the district had cell phone connectivity, though with variation in the network operator available. For those facilities without electricity, a solar charger was provided with the tablet. In addition to the base maps, the primary analysis presented in this paper uses two data sources: (1) paper logs for both T-HIT and control sites and (2) T-HIT entries.

Paper registries are the standard of care at health facilities across Tanzania and much of sub-Saharan Africa; health workers handwrite data for all mothers receiving care at the time of a visit in facility logs and on the maternal health card. Study research assistants collected data from all fourteen intervention and control sites relevant to PMTCT outcomes from patient antenatal registries for a 20-week (5-month) period before initiation of T-HIT and for the 12-week (3-month) intervention period when T-HIT was being used. Data were also collected through the T-HIT system for the seven sites from February 23, 2015 to May 23, 2015.

Health workers at T-HIT sites continued maintaining handwritten records to adhere to the standard of care, as well as documenting care delivery via the T-HIT tablets. A unique identification number was generated for each patient to protect privacy and confidentiality.

### Data Analysis

This analysis focuses on documenting HIV testing in the intervention communities, as well as comparison of the total number of antenatal visits, HIV tests, and women testing positive for HIV over time in T-HIT and non-T-HIT sites. Although T-HIT captured antenatal, delivery, and postnatal data, the modeling only utilized the antenatal data as this corresponded to the antenatal paper records collected at intervention and control sites. Furthermore, we utilized the total visits from the T-HIT system, rather than unique patient visits, in the analysis because of an inability to link records in the paper patient logs. Linking patient data in the facility logs would require a manual record-by-record evaluation, looking for the name of each patient in every subsequent record during the pre- and postintervention phases, which was not feasible. Importantly, in the context of documenting trialability, the total number of visits reflects workload.

The analyses included descriptive data summary and mapping along with multivariate modeling. We generated total numbers from the T-HIT system to illustrate the level of productivity. These data were, in turn, mapped using *ArcGIS version 10.2* (Esri, Redlands, CA) geographic information system (GIS) software to offer visual depictions of HIV incidence and testing distribution among the T-HIT intervention sites. Generalized estimating equations (GEEs) in *SAS version 9.4* (SAS Institute Inc, Cary, NC) were used to model the effect of time (week number), phase (preintervention vs postintervention), and record type (T-HIT electronic, T-HIT paper, or non-T-HIT paper). A weekly visits rate ratio, as well as odds ratio was used to determine if there were greater odds of being seen in T-HIT or non-T-HIT sites.

Segmented mixed-model Poisson regression with an autoregressive (AR[1]) structure for repeated measures on facilities over weeks were used, trying several functional forms by week, including linear, quadratic, cubic, and categorical. In no case was the time effect significant, so the simplest functional form, linear, was used in the final model. Additionally, interaction terms were initially included to allow the effect of time to vary by phase, the effect of group to vary by phase, and the effect of time to vary by group and phase (the group×phase×week interaction). These interactions were removed from the model one at a time using backwards elimination. In the end, there were no significant interactions, although the week×phase interaction remained in the model to preserve the segmented nature of the model.

GEEs were also used to model the effect of time (week number), phase (pre intervention vs post intervention), and record type (T-HIT electronic, T-HIT paper, or Non-T-HIT paper) on the odds of (1) being tested for HIV and (2) testing positive for HIV using segmented mixed model logistic regression with an AR(1) structure for repeated measures on facilities over weeks. Again, several functional forms for week were tried, including linear, quadratic, cubic, and categorical. In no case was the time effect significant, so the simplest functional form, linear, was used in the final model. Additionally, interaction terms were initially included to allow the effect of time to vary by phase, the effect of group to vary by phase, and the effect of time to vary by group and phase (the group×phase×week interaction) for each model. These interactions were removed from the model one at a time using backwards elimination. In the end, there were no significant interactions, although the week×phase interaction remained in the model to preserve the segmented nature of the models.

### Ethical Statement

Ethical approval was obtained from the Conjoint Catholic University of Health and Allied Sciences and Bugando Medical Centre Research Review and Ethics Committee (Ref. *CREC/051/2013*), the Tanzania National Institute for Medical Research (*NIMR/HQ/R.8a/Vol.IX/1662*), and the Colorado Multiple Institutional Review Board (Protocol 13-2166). Permission to conduct research in Tanzania was also obtained from the Tanzania Commission for Science and Technology and regional and district authorities in Mwanza and Misungwi, respectively. Health worker participation in the study was voluntary, and all who were approached agreed to take part.

## Results

Table 1 presents descriptive data on facilities and mean number of visits, HIV tests, and numbers testing positive for HIV from the T-HIT electronic records. Health workers used T-HIT to document 1594 antenatal visits across 1530 unique patients, with 558 of these visits at the district hospital. There were between 69 and 226 antenatal visits to dispensaries and health centers and an average of 16.27 antenatal visits each week across all T-HIT sites. Almost all women (96.01%, 1469/1530) had only one antenatal visit, and only 56 women in the pilot had more than one antenatal visit. We documented outcomes from 695 HIV tests that took place during an antenatal visit in the pilot period; of these, there were 55 women who tested positive (3.59% of the 1530 unique women seen during the pilot). Importantly, T-HIT documented if a health worker was unable to conduct an HIV test, most likely because of a lack of reagent. T-HIT documented 103 women for whom health workers were unable to successfully complete an HIV test antenatally (6.73%, 103/1530 of the unique women seen). Those facilities with five or less visits are not reported for confidentiality purposes, although the data were included in the model.

Paper records at the T-HIT sites recorded significantly fewer antenatal visits during the same period (N=879), though similarly proportionately lower at all facilities except Misasi Health Centre, which was the only site to record a higher number in the paper records. The total number of HIV tests (N=486) and positive HIV results (N=22) were also substantially lower than the T-HIT electronic records. Table 2 presents the paper records for the intervention period for the non-T-HIT control sites. The total number of visits (N=866), HIV tests (N=343), and HIV positive results (N=25) are comparable between the paper control and intervention records.

**Table 1:** Antenatal visit information at Tanzania Health Information Technology (T-HIT) health facility sites.

Health facility sites		Misungwi District Hospital	Misasi Health Center	Mwawile Dispensary	Mbarika Health Center	Mondo Dispensary	Nguge Dispensary	Gambajiga Dispensary	Total T-HIT
**Electronic records**									
	Total visits	558	69	187	226	205	175	164	1594
	Mean per week (SD^a^)	40.6 (15.6)	4.9 (5.5)	13.4 (8.8)	16.1 (11.0)	14.6 (8.8)	12.5 (8.1)	11.7 (6.2)	16.27 (3.38)
	Total HIV tests	193	6	97	121	39	139	100	695
	Mean per week (SD)	14.1 (11.0)	0.46 (1.13)	6.9 (5.2)	9.0 (6.7)	2.5 (3.6)	10.0 (7.8)	7.7 (4.1)	7.2 (3.2)
	Total unable to complete an HIV test	23	—^b^	24	9	34	10	0	103
	Mean per week (SD)	1.6 (3.5)	—	1.7 (3.6)	0.6 (1.2)	2.4 (2.7)	0.7 (2.2)	0 (0)	1.1 (1.4)
	Total testing HIV+	26	—	10	—	0	6	—	55
	Mean per week (SD)	1.9 (1.3)	—	0.7 (1.4)	—	0.0 (0.0)	0.4 (0.5)	—	0.6 (0.5)
**Paper records**									
	Total visits	307	258	79	104	34	65	32	879
	Mean per week (SD)	29.63 (9.49)	22.63 (7.71)	8.07 (3.73)	9.81 (4.9)	3.00 (1.92)	6.64 (2.65)	3.92 (2.43)	12.50 (11)
	Total HIV tests	202	56	64	68	6	58	32	486
	Mean per week (SD)	22.50 (10.58)	4.81 (6.75)	6.47 (4.03)	6.81 (5.74)	0.64 (1.34)	6.14 (2.93)	3.85 (2.44)	7.58 (8.75)
	Total testing HIV+	8	—	7	—	0	—	—	22
	Mean per week (SD)	0.69 (0.95)	—	0.73 (0.88)	—	0.00 (0)	—	—	0.35 (0.69)

^a^SD: standard deviation.

^b^Five or less visits reported; totals were included in the model.

**Table 2:** Antenatal visit information at comparison health facility sites, intervention phase.

Health facility sites		Bukumbi Mission Hospital	Busongo Health Center	Igokelo Dispensary	Koromije Health Center	Lubiri Dispensary	Nyamijundu Dispensary	Nyang homango Dispensary	Total paper cohort
**Paper records**									
	Total visits	178	137	94)	179	70	131	77	866
	Mean per week (SD^a^)	10.81 (3.67)	10.60 (3.25)	5.94 (3.51)	16.1 (11.0)	5.57 (3.41)	12.5 (8.1)	6.29 (3.65)	8.91 (5.06)
	Total HIV tests	95	34	14	71	27	47	55	343
	Mean per week (SD)	7.69 (3.65)	1.93 (3.47)	0.19 (0.4)	4.06 (5.18)	2.00 (2.88)	10.0 (7.8)	8.08 (5.33)	4.20 (4.65)
	Total testing HIV+	21	—^b^	—	6	0	—	—	25
	Mean per week (SD)	0.56 (0.89)	—	—	0.31 (0.79)	0.00 (0)	—	—	0.24 (0.6)

^a^SD: standard deviation.

^b^Five or less visits reported; totals were included in the model.

**Figure 2:**
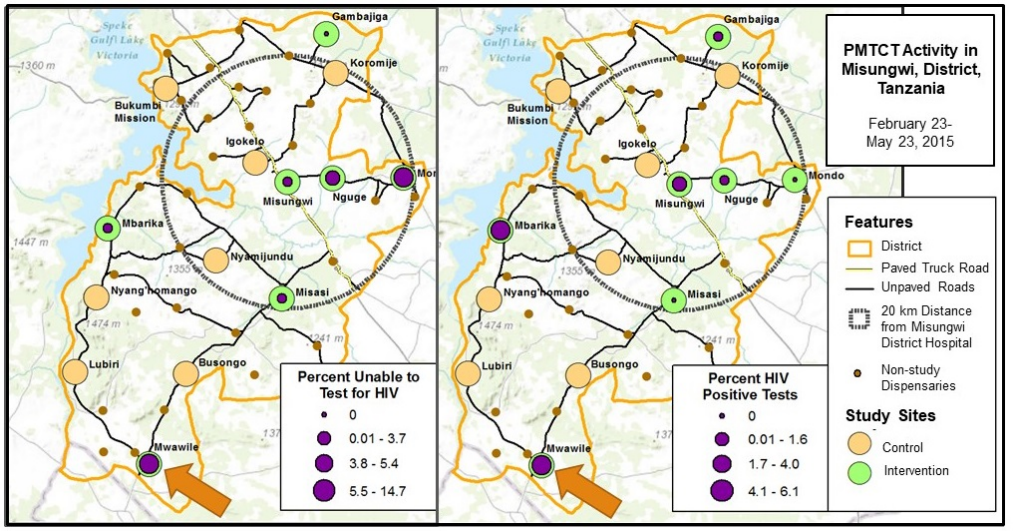
Maps demonstrating testing access and HIV incidence at Tanzania Health Information Technology (T-HIT) study sites.

Figure 2 offers a mapped depiction of the distribution of intervention and control sites, along with HIV incidence and testing patterns in intervention sites. The small orange-brown dots represent the location of 28 dispensaries not randomized into the study. The large orange circles are the control sites and the green circles, the intervention sites. The graduated purple circles inside the intervention sites show the percentage of HIV positive tests captured in the T-HIT system on the right map and the percentage of unable to test. Bukumbi Mission and Misungwi are the two hospitals and Misassi, Koromije, Mbarika, and Busongo the health centers. As highlighted with an arrow at the bottom of each map, one site, Mwawile Dispensary, emerged as having both a high number of people unable to test for HIV (shown on the left) and simultaneously a high incidence of HIV among women testing (shown on the right). Further investigation with clinic staff in Mwawile Dispensary revealed that the clinic ran out of reagent for testing during this period, and health workers were unable to complete HIV testing in 12.8% (24/187) of cases.

Table 3 shows rate ratios for total antenatal visits and odds ratios for testing and testing positive for HIV, comparing T-HIT electronic records with paper records at non-T-HIT sites and comparing visits in the preintervention phase to the intervention phase. The number of *weekly antenatal visits* (patients seen) did not differ significantly from week-to-week before the intervention (*P*=.14). This weekly rate of change did not differ significantly in the postimplementation phase with respect to the preimplementation phase (*P*=.83), and the implementation itself did not cause any change in the number of patients seen per week (*P*=.75).

The odds of *being tested* for HIV did not differ significantly from week-to-week before the intervention (Table 2, *P*=.35). We observed a drop in HIV testing comparing the preintervention with the postintervention period, where women were 0.06 times as likely to be tested after the pilot implementation as before (*P*=.01).

**Figure 3:**
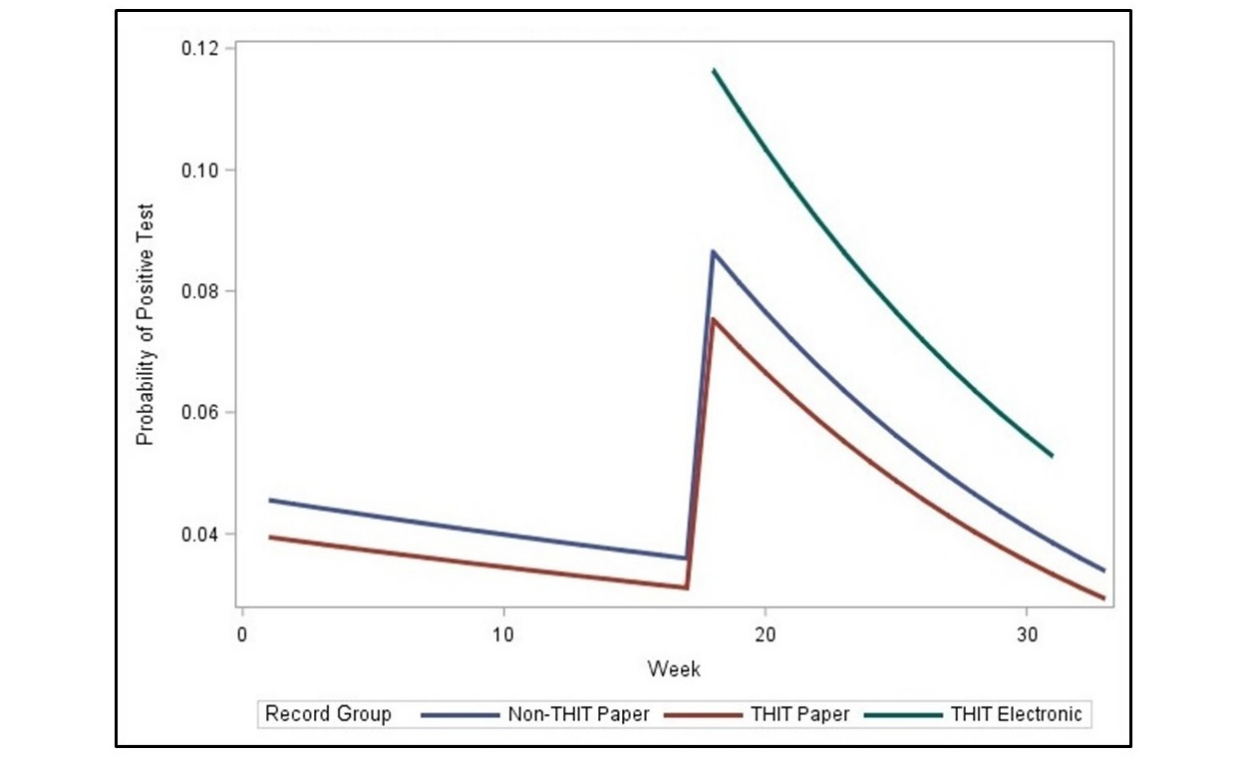
Odds of testing positive for HIV.

The odds of *testing positive* for HIV did not differ significantly from week-to-week before the intervention (*P*=.55). Specifically, the odds of testing positive for HIV dropped 2% (95% CI: a drop of 6% to an increase of 4%). However, the weekly rate of change did differ significantly in the postimplementation phase with respect to the preimplementation phase (*P*=.04). The weekly rate of change in the postimplementation phase was 5% lower than that in the preimplementation phase (95% CI: 9% decrease to 0% increase), giving a significant postintervention weekly rate of change (*P*=.004), with the odds of testing positive dropping by 6% (95% CI: 11% drop to 2% drop) in the postimplementation phase. This is represented graphically in Figure 3.

**Table 3:** Model outcomes for rate and odds ratios of patient visits, tested for HIV, and tested positive for HIV; Tanzania Health Information Technology (T-HIT) electronic records compared with paper records in T-HIT sites and comparison sites.

Variable and category			Rate ratio (95% CI)	*P* value
**Rate ratios for the number of patients seen weekly**				
	Week		0.99 (0.97-1.00)	.14
	**Record group**			.20^a^
		T-HIT electronic versus non-T-HIT paper (week 18)	1.52 (0.87-2.66)	.14
		T-HIT electronic versus T-HIT paper (week 18)	1.14 (0.67-1.95)	.63
		T-HIT paper versus non-T-HIT paper (week 18)	1.11 (0.61-2.01)	.74
	Phase			
		Post intervention versus pre intervention	1.04 (0.71-1.53)	.83
	Week×phase interaction			
		Post intervention versus pre intervention	1.00 (0.98-1.03)	.75
**Odds ratios for the number of patients who were tested for HIV**				
	Week		0.95 (0.86-1.06)	.35
	**Record group**			.77^a^
		T-HIT electronic versus non-T-HIT paper (week 18)	0.89 (0.27-2.90)	.85
		T-HIT electronic versus T-HIT paper (week 18)	0.65 (0.24-1.79)	.41
		T-HIT paper versus non-T-HIT paper (week 18)	1.28 (0.26-6.16)	.76
	Phase			
		Post intervention versus pre intervention	0.06 (0.01-0.29)	.001
	Week×phase interaction			
		Post intervention versus pre intervention	1.10 (0.97-1.23)	.13
**Odds ratios for the number of those tested who were positive for HIV**				
	**Week×phase interaction**			
		Post intervention versus pre intervention	0.95 (0.91-1.00)	.04
		Preintervention effect of week	0.98 (0.94-1.04)	.55
		Postintervention effect of week	0.94 (0.89-0.98)	.004
	**Record group**			.69^a^
		T-HIT electronic versus non-T-HIT paper (week 18)	0.67 (0.24-1.82)	.43
		T-HIT electronic versus T-HIT paper (week 18)	0.78 (0.23-2.63)	.68
		T-HIT paper versus non-T-HIT paper (week 18)	0.41 (0.19-0.89)	.02
	Phase			
		Post intervention versus pre intervention	6.43 (2.26-18.35)	.001

^a^Type 3 *P* values.

## Discussion

### Principal Findings

Although there is high acceptability of mHealth solutions in low and middle-income countries (LMIC), evidence for intervention efficacy is limited in LMIC settings [14]. Overall, the evidence from this study suggests that health workers can, and will, use the T-HIT system to record PMTCT visit data. Health workers in the T-HIT hospital, health centers, and dispensaries all used the system without overt challenges. Due to strong indications for relative advantage and feasibility, T-HIT has the potential for scaling. Furthermore, T-HIT may well contribute to increases in PMTCT visits if the quality of care by health workers improves through a system that supports decision making. This alone is a reason to implement and test T-HIT efficacy on a larger scale. We also anticipate dividends beyond the capacity to document in this pilot that can be realized in a larger trial of T-HIT. For example, more immediate information sharing from the T-HIT system to the district hospital could better distribute reagent for HIV testing, shorten time frames to distribute ARV, and better plan for hospital delivery and infant care for HIV positive mothers.

The data suggest that the intervention and control sites were comparable in terms of overall visits and HIV testing for mothers before and during the study period. The T-HIT system captured potentially critical data on the inability to test for HIV, revealing the need for redistribution of HIV reagent much more immediate at some sites. This is a particularly pressing need for clinics with higher HIV incidence, as most women do not return for follow-up antenatal visits when a follow-up test could occur.

The integration of data across visits for mothers is another critical contribution of an electronic data entry mHealth solution. In the paper record system, there are separate logs for antenatal visits, delivery visits, and postnatal visits and yet another record kept by the mother in the form of the MCH card. The T-HIT system has the potential for linking a patient’s record across numerous visits. In this way, a woman’s health status can be tracked, including risk factors and HIV testing status throughout pregnancy, delivery, and postnatal care. This, in turn, greatly improves opportunities for continuity of care. In an expanded and longer T-HIT trial, monitoring patient-level visits in combination with improved care reinforced by T-HIT decision-support would likely yield an improvement in the number of pre- and postnatal visits, along with an increase in hospital delivery numbers.

A decline in testing for HIV was observed when comparing the intervention period to the preintervention period across all sites, suggesting something other than the newly introduced T-HIT system affected declines in HIV testing. Despite this decline in testing, there was an overall increase in the number of women testing positive for HIV during the pilot study implementation period compared with the preimplementation, with no difference in intervention compared with control sites. This, at least, suggests that whatever the background driver was, the trend was captured in both the paper logs and T-HIT, indicating health workers utilized the interface successfully and these data were appropriately captured by the system, providing evidence for feasibility. In a longer trial, we also posit an improvement for HIV testing capacity and volume resulting from the ability of the health delivery system to effectively respond to real-time stock outages such as HIV reagent.

The odds of testing positive for HIV suggest that women in T-HIT were not more likely to have testing in this short pilot; this suggests an uptick in the numbers of women documented with HIV immediately after the intervention was implemented (regardless of whether it was documented on a paper log or the T-HIT system) that reverted to preintervention levels over time. This may represent a Hawthorne effect, or possibly a temporal factor such as weather or seasonal events. At a minimum, this also provides additional evidence in support of feasibility of the mHealth solution.

T-HIT consistently recorded a higher number of visits, HIV tests, and HIV positive results. We hypothesize that this is quite possibly because the T-HIT system was capturing data that would be found in separate paper log books. Consequently, T-HIT might be already exhibiting the capability for integrating data sources. However, this could also be a result of health workers entering data after the visit, or entering additional data to show an increase of activity knowing that the totals were being displayed in near real time. However, it would be challenging to consistently adjust over 3 months and similarly across facilities. In one instance, Misasi Health Centre entered far fewer records than would be expected entered into T-HIT based both on a comparison to paper records at that site and to the activity at other health centers. Thus, this is likely evidence of a facility that did not fully adopt or implement the T-HIT system with regularity.

We have yet to see widespread adoption of mHealth solutions for care delivery. This suggests incentives are needed to facilitate adoption and use that are targeted at various components of the health care system. For example, incentives can be aimed at the health worker through training or monetary compensation. Perhaps one of the most important incentives is for the system to have perceived usefulness in terms of increasing confidence for providing care. Additionally, policies that obligate use can be established at the systems level. However, before policies that require use of mHealth tools can be realistically established, a careful assessment is needed to ensure organizational readiness to train users and offer technical support for devices and data management. Data quality (completeness and accuracy) issues persist for PMTCT, and although opportunities for improvement are complex, perceived usefulness along with support and supervision are necessary to produce reliable and informative [15]. Long-term sustainability can likely not rely on remuneration for users in low-resource settings and so, multi-faceted approaches for implementation and scaling will be required.

As implementation of mHealth solutions for care delivery increases, a critical consideration of costs associated with technology infrastructure will be required to evaluate whether investment in this infrastructure is warranted. Existing more “low-tech” approaches to data collection, such as pen and paper, may be sufficient. However, if decision makers determine that infrastructural investment in technology for health care delivery is appropriate, then attention to multiple areas to maximize this investment is needed. Many careful considerations are necessary, including technology or equipment choices (computers, servers, phones, and tablets), sufficient staff who can program and maintain such equipment, development of protocols and training programs for health care workers to effectively use technology, development of policies and incentives to motivate use, and attention to regular process evaluations to ensure efficiency and quality in data collection and communication.

### Conclusions

This pilot study documented the successful use of the tablet-based mHealth T-HIT system, demonstrated its feasibility for the effective use by health workers, and illustrated the capacity for capturing, facilitating, and monitoring HIV testing and new infections for PMTCT. Furthermore, findings indicate T-HIT offers a potentially scalable mHealth solution for PMTCT data collection to facilitate decision support in resource-scarce settings. Importantly, although the T-HIT system was designed to directly address PMTCT, it could be easily adapted to facilitate monitoring and integrated care delivery for any number of priority health concerns such as antenatal or postnatal care, tuberculosis care, malaria, and chronic conditions.

## Abbreviations

ANC: antenatal care
AR: autoregressive
ARV: antiretroviral
GEE: generalized estimating equation
GIS: geographic information system
LMIC: low and middle-income countries
MCH: maternal and child health
mHealth: mobile health
PMTCT: prevention of mother-to-child transmission
T-HIT: Tanzania Health Information Technology
WHO: World Health Organization

## References

[1] (2016). World Health Organization.

[2] Joint United Nations Programme on HIV/AIDS (2013). Global Report: UNAIDS report on the global AIDS epidemic 2013.

[3] Manyahi J, Jullu BS, Abuya MI, Juma J, Ndayongeje J, Kilama B, Sambu V, Nondi J, Rabiel B, Somi G, Matee MI (2015). Prevalence of HIV and syphilis infections among pregnant women attending antenatal clinics in Tanzania, 2011. BMC Public Health.

[4] World Health Organization (2017). Avert.

[5] Tanzania Commission for AIDS (TACAIDS) (2013). Country Planning Cycle Database.

[6] Tanzania Commission for AIDS (TACAIDS), Zanzibar AIDS Comission (ZAC), National Bureau of Statistics (NBS), Office of Chief Government Statistician (OCGS), ICF International (2013). Dhsprogram.

[7] International Telecommunication Union (ITU) (2015). Measuring the Information Society Report.

[8] Betjemen TJ, Sogoian SE, Foran MP (2013). mHealth in Sub-Saharan Africa. Int J Telemed Appl.

[9] US Department of Health and Human Services (2014). Health Resources and Services Administration.

[10] Gurman TA, Rubin SE, Roess AA (2012). Effectiveness of mHealth behavior change communication interventions in developing countries: a systematic review of the literature. J Health Commun.

[11] Kallander K, Tibenderana JK, Akpogheneta OJ, Strachan DL, Hill Z, ten Asbroek AHA, Conteh L, Kirkwood BR, Meek SR (2013). Mobile health (mHealth) approaches and lessons for increased performance and retention of community health workers in low and middle income countries: a review. J Med Internet Res.

[12] Bull S, Ezeanochie N (2016). From Foucault to Freire through Facebook: toward an integrated theory of mHealth. Health Educ Behav.

[13] White A, Thomas DS, Ezeanochie N, Bull S (2016). Health worker mHealth utilization: a systematic review. Comput Inform Nurs.

[14] Hurt K, Walker RJ, Campbell JA, Egede LE (2016). mHealth interventions in low and middle-income countries: a systematic review. Glob J Health Sci.

[15] Mate KS, Bennett B, Mphatswe W, Barker P, Rollins N (2009). Challenges for routine health system data management in a large public programme to prevent mother-to-child HIV transmission in South Africa. PLoS One.

